# Equivalent volume: study in subjects with chronic otitis media

**DOI:** 10.1016/S1808-8694(15)31270-2

**Published:** 2015-10-20

**Authors:** Ana P.T. Alencar, Maria C.M. Iório, Douglas S. Morales

**Affiliations:** 1 Speech and Hearing Therapist, Master in Human Communication Disorders (UNIFESP-EPM).; 2 Speech and Hearing Therapist, Ph.D. in Human Communication Disorders (UNIFESP-EPM).; 3 Otorhinolaryngologist, Master in Otorhinolaryngology, (UNIFESP-EPM).; Affiliation: Federal University of Sao Paulo – Escola Paulista de Medicina.

**Keywords:** chronic otitis media, acoustic impedance tests

## Abstract

The equivalent ear canal volume ranges from 0.3ml to 1.0ml in children and from 0.65 to 1.75ml in adults. In subjects with chronic otitis media these values can be different, according to the disease status. **Aim:** To study the equivalent ear canal volume in 52 ears of patients with chronic otitis media with and without active infection. **Study design:** clinical prospective with transversal cohort. **Material and Method:** The equivalent ear canal volume was obtained from 52 ears diagnosed with chronic otitis media with and without active infection and in age and gender matched control group. The study group with active infection was evaluated before and after clinical treatment. **Results:** Equivalent ear canal volume mean for the studied groups with and without infection and for the control group was 2.86ml; 1.42ml and 0.80ml, respectively. The equivalent ear canal volume mean for the study group with infection prior and post clinical treatment was 1.42ml and 1.82ml, respectively. **Conclusions:** The Equivalent ear canal volume mean was higher in patients with Chronic Otitis Media. We did not observe variation of equivalent ear canal volume before and after clinical treatment.

## INTRODUCTION

Equivalent volume of the external acoustic canal is one of the measures of tympanometry, which is conventionally subtracted from total admittance measures to provide an estimate of the middle ear admittance. It may be obtained by the introduction of a relatively high (+200 daPa) or low (-400 daPa) air pressure in the external auditory canal. In such pressures, impedance of middle ear and tympanic membrane, for clinical purposes, is infinitely high and admittance is virtually zero. Thus, immittance is measured only by the volume of air placed in the external acoustic canal between the tip of the probe and the tympanic membrane.^1^

The equivalent external acoustic canal volume in subjects with normal tympanic membrane ranges from 0.3ml to 1.0ml in children and 0.65ml to 1.75ml in adults and normally they are smaller in women than in men ^1^.

In subjects with chronic otitis media, in whom there are tympanic membrane perforation, this measure can be affected depending on the condition. In such cases, the volume obtained with the introduction of high or low pressures in the acoustic canal should be defined as the equivalent ear volume, given that it includes, in addition to the external acoustic canal, also the space of the middle ear, antrum and air cells of the mastoid.

According to some authors ^2^, in the presence of a flat tympanogram, the equivalent ear volume may be useful to detect tympanic membrane perforations and despite the fact that the normal volume does not exclude it, the presence of flat curve and high volume may evidence it.

Given that there are no reports in the Brazilian literature about the use of equivalent volume measures in chronic otitis media patients, the purpose of the present study was to analyze the equivalent ear volume in the presence of a flat tympanogram trying to check whether there are differences in equivalent volume in patients with perforation of tympanic membrane followed or not by active infectious process.

## MATERIAL AND METHOD

The present study was approved by the Research Ethics Committee, UNIFESP/EPM, process nº 0033/02.

The collected material comprised the measurement of equivalent volume (VEQ) in 52 ears with chronic otitis media with suppuration (COMS) of 43 subjects followed up in the Ambulatory of Otorhinolaryngology, Hospital Geral de Pirajussara, in the period between March and September 2002. The age range of the studied population was between 8 and 60 years, 27 female and 16 male subjects. We selected only subjects whose affected ear had never been submitted to otological surgical treatment and had no signs of cholesteatoma or damage to the ossicle chain.

The recruitment of subjects with COMS was performed by the Otorhinolaryngologist during the outpatient visit, by means of the performance of detailed otoscopy to reach a reliable diagnosis of the disease. The ENT assessment enabled the classification of ears with COMS forming two studied groups.

The group named GESI (Studied Group without Infection) comprised 28 ears with diagnosis of chronic otitis media with suppuration, without signs of active infection. The group manifested tympanic membrane perforation, dry or wet cavity with white or transparent secretion and normal tympanic cavity mucosa. The second group named GEI (study group with infection) had 24 ears with diagnosis of chronic otitis media with suppuration and signs of active infection, manifesting perforation of tympanic membrane, tympanic cavity with yellow or green secretion and mucosa of the tympanic membrane with hyperemia or polypoid aspect.

To standardize the care provided, a protocol of assessment of patients with COMS was previously prepared by the ENT physician and by the responsible audiologist and comprised: history, otoscopy, diagnostic hypothesis by the Otorhinolaryngologist and equivalent ear volume measures.

To enable comparisons, we created a control group formed by 43 subjects (52 ears) with normal tympanic membrane Hospital Geral de Pirajussara, between May 2001 and September 2002. We selected only subjects whose history, otoscopy and audiological assessment (pure tone audiometry, speech audiometry and impedanciometry) did not show any middle ear affection.

Right after otoscopy, subjects with COMS selected by the Otorhinolaryngologist were submitted to a brief anamnesis, referring to the otological history, and next the audiological assessment which comprised pure tone and speech audiometry and ear equivalent volume (VEQ). Patients in the GEI group were assessed at two different points: before and after treatment.

Audiological assessment comprised pure tone and speech audiometry.

Pure tone audiometry was performed with air bone conduction in all subjects in frequencies 250 Hz to 8kHz. In those that presented thresholds above 25 dB HL (normal range), we collected also bone conduction thresholds in frequencies 500Hz to 4000Hz. We also investigated the thresholds of speech recognition with dissyllable words and percentage index of speech recognition with monosyllable words ^3^. To perform the audiometry, we used audiometer model AC 33 brand Interacoustics, with supraural phone TDH-39 and pads MX-41, according to the calibration norms ANSI, 1969.

Data obtained in these exams were not used in the study, but they were performed for being part of the routine assessment.

The Equivalent ear volume was obtained by completely sealing the external acoustic canal of the patients with a probe protected with a flexible material tip, introducing through it a pressure of + 200 daPa in the EAC (external acoustic canal)^5^, ^6^, ^7^. Next, we checked the equivalent volume VEQ in the complacency screen. We used impedanciometer model AZ7 brand Interacoustics, with test probe of 226 Hz, and supraural phone TDH-39 and pads MX-41.

Results were obtained and recorded in the subjects’ protocol.

Some patients required clinical treatment, that is, they had active infectious process and were again submitted to the same protocol after the clinical treatment; there were 21 subjects (24 ears) initially accessed, 17 patients (18 ears) came back for the assessment after treatment.

Thus, right after the first assessment, subjects received free samples of the drug to apply the treatment at home and after it, they came back to the ambulatory where they were submitted to new otoscopy and the same protocol used in the first assessment, which comprised new measurements of VEQ.

It is important to point out that for the Equivalent volume, flexible material tips were identical to those used in the first assessment.

All patients were submitted to the same treatment: application of three drops of Ciprofloxacin 0.3% (sterile otological solution) in the affected ear, three times a day, for a period that ranged from 7 to 10 days, according to the indication of the ENT physician.

The choice of medication (active substance) was made by the ENT physician.

Equivalent volumes in the studied groups (GESI and GEI) and in the control group were statistically compared and analyzed. For the analysis of these findings, we used descriptive and conclusive statistical techniques, parametric or non-parametric. We applied the following tests:

1. Comparison of Univariate analysis (ANOVA) to compare VEQ in the studied groups GEI and GESI and in the control group.
2. Wilcoxon test for the comparison of VEQ in GEI before and after clinical treatment.

In all cases, level of significance adopted was 0.05 or 5% (x< 5%), and the significant values were marked with an asterisk.

## RESULTS

**Figure 1:**
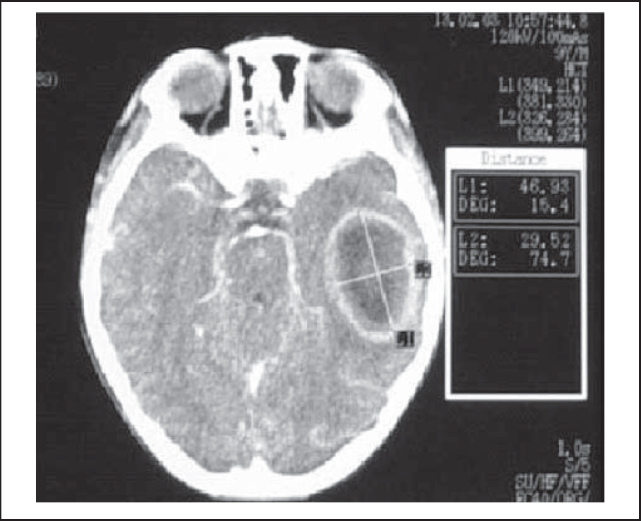
Graph showing minimum, median and maximum values of equivalent volume in ml of ears with COMS with and without infection and the control group.

After studying VEQ in 52 ears and their respective controls (Annex 1), we calculated the arithmetic mean of VEQ of the ears in the study group GESI and GEI and in the control group and we performed a comparative study of these measures between the groups, using the Univariate Analysis ANOVA test. These data may be observed in Table 1 and in the Graph that is presented in Figure 1.

**Annex 1:** Measurements of equivalent volume in ml in the ears of the studies group with infection (VEQGEI) and their controls (VEQConGEI) and in the group without infection (VEQGESI) and their controls (VEQConGESI).

Veq GESI	Veq ConGESI	Veq GEI	VeqConGEI
1,0	1,2	1,3	1,0
4,4	0,7	0,5	0,8
3,0	0,7	0,9	0,6
5,0	0,7	0,8	0,6
2,4	0,5	5	0,8
1,2	0,5	1,2	0,9
0,7	0,9	1,5	1,0
2,2	0,9	0,7	0,6
5,0	0,7	1,1	0,6
2,3	0,8	0,8	0,8
0,6	0,8	5,0	1,0
5,0	0,7	1,4	1,1
5,0	1,0	0,8	1,1
2,1	0,7	0,8	0,7
3,0	0,8	0,6	0,6
1,7	0,9	0,7	0,9
2,1	0,6	1,2	0,6
0,6	1,0	0,5	0,7
4,2	0,7	1,0	0,8
5,0	0,8	0,8	0,7
3,8	0,6	5,0	0,8
5,0	0,5	0,4	0,7
2,1	0,8	1,4	1,1
0,8	1,4	0,6	1,0
2,8	1,0		
5,0	0,6		
1,8	0,7		
2,2	0,7

**Table 1:** Arithmetic mean and variance of equivalent volume in ml of ears in the group without infection (GESI) and with infection (GEI) and the respective control groups and p-value (univariate analysis).

	GESI		GEI	
	Study	Control	Study	Control
Mean	2,86	0,78	1,42	0,81
Variance	2,52	0,04	2,01	0,03
Size (N)	28	28	24	24

P-Value	< 0,001*		0,044*

**Figure 2:**
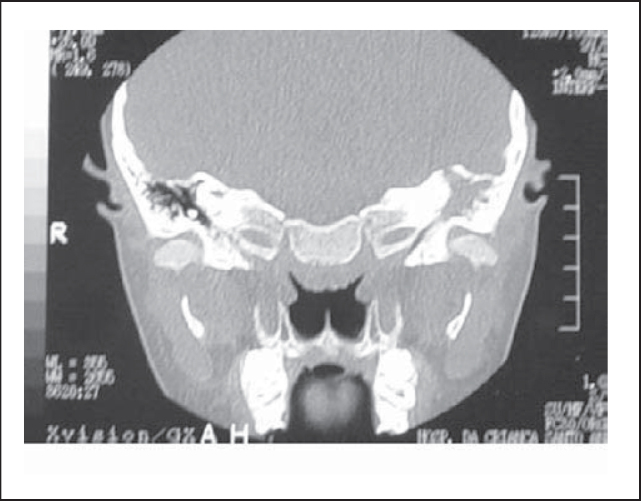
Graph showing minimum, median and maximum values of Equivalent volume in ml in ears of the studied group with infection before and after clinical treatment.

Next, we performed a study of VEQ in 18 ears of the GEI group before and after clinical treatment (Annex 2) and based on it, we calculated the arithmetic mean of VEQ; using Wilcoxon test, we compared the measurements obtained in the two groups, whose results are presented in Table 2 and Figure 2.

**Annex 2:** Measures of equivalent volume in ml of 24 ears in the studied group with infection (GEI), pre-treatment and 18 ears of the same group after clinical treatment.

VEQ GEI PRE-TTO	VEQGEI POST-TTO
1,3	1,7
0,5	0,8
0,9	1,5
0,8	0,8
5	5
1,2	1,3
1,1*	1,4
0,8*	1
5	5
1,4	1,6
0,8	1
0,8	1,4
0,7	0,9
0,5	0,6
0,8	1,4
5	5
1,4	1,5
0,6	0,8

**Table 2:** Mean arithmetic and variance of equivalent volume in ml of ears of the group with infection before and after clinical treatment and result of Wilcoxon test (p-value).

	VEQ GEI PRE- TTO	VEQ GEIPOST- TTO
Mean	1,42	1,82
Variance	2,01	2,25
Size (N)	24	18

P-Value	0,108

## DISCUSSION

In the analysis of Equivalent Volume (VEQ) of the studied group without infection GESI (2.86ml) and the control group (0.78ml), we observed that VEQ for GESI group was significantly higher than for the control group (Table 1), as observed in previous studies^7^.

Thus, the statistically significant difference obtained between VEQ for the ears with COMS without infection and the ears with normal TM was also observed in studies that emphasized that VEQ is a good predictor of TM perforation in the absence of active middle ear mucosa infectious process, and that an abnormally large volume suggests TM perforation and normal middle ear/mastoid space 2, 8.

The analysis of mean VEQ in the studied group with infection GEI (1.42ml) and in the control group (0.81ml) has also revealed statistically significant difference (p< 0.044), despite the fact that the difference is close to the level of 0.05 (Table 1). Thus, we obtained larger volumes in the ears in the studied group GEI than in the control group. The findings of these study disagreed with what the literature brings, which revealed that cases of COMS in the presence of active infection have means of equivalent volume that are the same or very close to the volumes obtained in ears with normal TM 2, 8; in addition, there are studies that found smaller volumes for ears with active infections process than in normal TM cases without previous history of otological disease ^9^.

We also noticed statistically significant difference between VEQ of the groups GESI and GEI (Table 1), and the volume obtained for the group GESI (2.86ml) was higher than for group GEI (1.42), that is, in the absence of active infectious process (ME mucosa in good conditions), we reached an average higher mean VEQ than in ears with active infectious process, in agreement with previous studies 2, 8, 9.

It is interesting to point out that VEQ behavior was different in the studied groups. We observed that in GEI (with infection) and for the control group there was no significant variation between volumes obtained in each group separately. For GEI, values were within 0.4ml and 1.5ml, with only three isolated values in 5.0ml. For the control group, values of VEQ were within 0.5ml and 1.4ml, and 79% of the values were approximately smaller than 1.0ml. For the studied group GESI (without infection), there was major variation in volumes, and the smallest value found was 0.6ml and the largest was 5.0ml, but approximately 70% of the volumes were above 2.0ml (Figure 1).

In the VEQ study of GEI group before and after clinical treatment, we could confirm that there was no statistically significant difference between them (Table 2). Even so, it is important to point out that after improvement of ME mucosa conditions (after clinical treatment) mean VEQ was greater than mean VEQ of ears assessed before treatment, showing a slight increase in variation of individual measured volumes (Figure 2).

It is important to emphasize that in two ears (shown with asterisks in Annex 2) they continued to present some signs of ME infection after clinical treatment. Nevertheless, both presented VEQ higher than in pre-treatment assessment, showing that even a slight improvement in ME conditions can modify VEQ.

Another factor to be highlighted is that four patients did not perform assessment after clinical treatment. This fact is normally observed in longitudinal studies, which many times may be hindered by such events.

We did not find in the investigated literature any authors that had performed similar investigations with VEQ assessment before and after treatment.

Considering these observation, there is the need for further studies to better understand pre and post-surgical treatment assessment of these patients to learn more about the application of the measure VEQ in patients with COMS.

## CONCLUSIONS

The critical analysis from the obtained results led us to conclude that:

1. Mean equivalent volume is higher in patients with suppurative chronic otitis media without and with active infection than in patients without perforations of the TM, respectively as 2.86ml; 1.42ml and 0.8ml;
2. There was no difference between equivalent volume before and after clinical treatment.
